# Finite Element Modeling of the Dynamic Response of Plywood

**DOI:** 10.3390/ma17174358

**Published:** 2024-09-03

**Authors:** Arkadiusz Charuk, Katarzyna Gawdzińska, Paweł Dunaj

**Affiliations:** 1 Faculty of Mechanical Engineering and Mechatronics, West Pomeranian University of Technology in Szczecin, al. Piastów 19, 70-310 Szczecin, Poland; arkadiusz.charuk@zut.edu.pl; 2 Marine Ship Repair Yard “Gryfia” J.S.C., Brdowska 12 Str., 71-700 Szczecin, Poland; 3 Faculty of Marine Engineering, Maritime University of Szczecin, 1-2 Wały Chrobrego St., 70-500 Szczecin, Poland; k.gawdzinska@pm.szczecin.pl

**Keywords:** wood composite panel, modal analysis, finite element method, plywood, sensitive analysis, engineered wood, wood, wood veneer

## Abstract

Modeling the dynamic properties of wood and wood-based composites is a challenging task due to naturally growing structure and moisture-dependent material properties. This paper presents the finite element modeling of plywood panels’ dynamic properties. Two panels differing in thickness were analyzed: (i) 18 mm and (ii) 27 mm. The developed models consisted of individual layers of wood, which were discretized using three-dimensional finite elements formulated using an orthotropic material model. The models were subjected to an updating procedure based on experimentally determined frequency response functions. As a result of a model updating relative errors for natural frequencies obtained numerically and experimentally were not exceeding 2.0%, on average 0.7% for 18 mm thick panel and not exceeding 2.6%, on average 1.5% for 27 mm thick panel. To prove the utility of the method and at the same time to validate it, a model of a cabinet was built, which was then subjected to experimental verification. In this case, average relative differences for natural frequencies of 6.6% were obtained.

## 1. Introduction

Wood and wood-based composites are widely used in industry due to their light weight, ease of processing, and strength properties [1]. However, the development of new wood products is a difficult task due to the naturally growing structure of wood and its moisture-dependent material properties [2], especially when different wood and wood-based materials are combined in one structure [3]. This forces the improvement of the existing and the development of new computational methods to predict the operational properties of structures made of wooden materials [4]. Very promising results have been obtained in the field of static properties [5], fracture [6], seismic performance [7] or moisture transport [8]; however, the issue of structural dynamics modeling still causes many problems [9].

Bolmsvik et al. [10] presented a finite element model of a lightweight wooden assembly consisting of wooden beams, gypsum boards, chipboards, and hard-processed fiberboards. The model was developed to predict the dynamic properties of the structure, i.e., natural frequencies, mode shapes, and frequency response functions. It was built in Abaqus (Vers. 6.11-2. Dassault Systèmes Simulia Corp. Providence, RI, USA) using 20-noded quadratic brick solid elements with full integration. The beams were modeled with an orthotropic material model, while the board elements were modeled using an isotropic one. The damping was modeled as a modal viscous damping superimposed on the entire structure. The parameters adopted for the description of the model were taken from the literature. The developed model was then subjected to experimental verification, after which the authors found that the obtained accuracy was unsatisfactory. Therefore, an analysis of the material parameter influence of dynamic properties was conducted, and on its basis, model updating was performed, giving satisfactory results. Two main conclusions were drawn: (i) the wood material properties had the largest influence on the behavior of the finite element model, and (ii) to improve the finite element model accuracy, component properties should be identified before assembling the structure.

Guan et al. [11] presented the modal analysis of three fiberboards and three particleboard panels with different dimensions. The analysis was based on the finite element models. The models were built using plate elements of the mixed interpolation of tensorial components and an orthotropic material model. The initial parameters describing orthotropic material models for analyzed panels were estimated on the basis of experimental modal analysis and static bending tests. Then, the first nine natural frequencies and mode shapes were calculated. The comparison of obtained results with the experimental ones showed full agreement in terms of mode shapes; however, the relative differences between natural frequencies were unsatisfactory. Therefore, the sensitivity analysis and a model updating procedure were conducted, giving the relative differences at a level not exceeding 6%. 

Paolini et al. [12] presented the modal and harmonic analysis of a cross-laminated timber structure. The structure consisted of a wooden wall and ceiling connected to each other by flat plate head screws for timber. The analysis performed in the study was based on the three-dimensional p-version of the finite element method. The finite element model consisted of successive cross-laminated timber layers represented by a single layer of finite elements with orthotropic material. Each layer consisted of hexahedral finite elements with shape functions of a second and fourth order depending on the element dimensions. Next, the connections on component and structure levels were modeled, i.e., between cross-laminated timber elements and a wall and a ceiling, respectively. After imposing boundary conditions, an eigenproblem was solved and natural frequencies and mode shapes for a free-hanging wall and the assembled test structure were determined. The results obtained were compared with experimental ones, and on this basis, the parameter identification procedure was performed. In the result precise prediction of cross-laminated timber structure dynamics was achieved giving accurate results in terms of natural frequencies, mode shapes and frequency response functions.

Manin et al. [13] presented a finite element analysis of a tennis racket blade composed of several wood layers. Two types of finite element models were developed. The first type considered individual layers of wood, which were discretized using two-dimensional multilayer composite parabolic shell elements. The second type represented the analyzed composite as a homogeneous structure discretized by two-dimensional or three-dimensional finite elements. Both types of models used orthotropic material. Next, a modal analysis was carried out considering two types of boundary conditions: (i) racket handle clamped and (ii) racket blade freely supported. The obtained results were verified experimentally, showing a good agreement. The authors concluded that the detailed composition of the plywood can be considered in the modeling, but it does not lead to better results than the modeling of one equivalent homogenized orthotropic wood layer.

In summary, it should be stated that the analyzed sources mostly indicate the need to fine-tune the model in order to obtain accurate results. Moreover, it can be noticed that the development of a reliable modal model (i.e., a set of natural frequencies and mode shapes) is mostly not so challenging [10,11,12,13]. The problem occurs when trying to predict the damping properties of wooden components, which is particularly reflected in the frequency response functions. Therefore, this paper presents a procedure for modeling the dynamic properties of plywood. Plywood is made of thin layers of wood called rotary veneers that are sequentially stacked perpendicular to each other. The purpose of such laying is to equalize physical and mechanical properties on the whole surface of the sheet, both lengthwise and crosswise [14]. This equalization is related to the fact that wood is an orthotropic material (it has unique and independent mechanical properties in the directions of three mutually perpendicular axes), which is related to nature and the process of growing trees that are the source of wood [15]. The inner layers of plywood are called core and are often made from veneers of less expensive woods, such as poplar. The outer layers are called face and back face, the material from which they are made determines the plywood classification. The individual layers are joined together with the use of urea-formaldehyde adhesives.

As part of the research, a finite element model of plywood was built, considering the material properties of its individual layers. The model updating procedure (supported by a sensitivity analysis) was then performed based on the results of the experimental modal analysis. The identified model was used to predict the dynamic properties of a complex structure.

## 2. Materials and Methods

### 2.1. Research Objects

**Figure 1:**
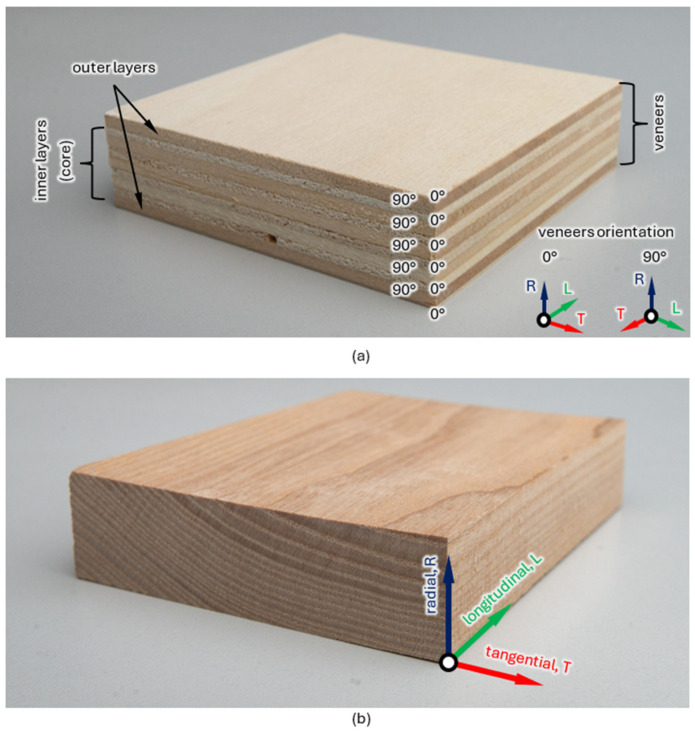
Analyzed plywood structure with sheets of veneers (**a**) and the wood structure (**b**).

The analyzed objects were poplar (*Populus tremula*) plywood (produced by Garnica Plywood Logroño (Garnica Plywood Logroño, Rioja, Spain) according to EN 14279:2004+A1:2009 standard [16]. The analyzed plywood is classified as B/BB, which indicates B grade top face and a BB grade bottom face. This means that the top face has a smooth, sanded finish with minimal imperfections, while the bottom face may have more noticeable knots or other natural blemishes. The tolerance for thickness given by the manufacturer was in accordance with EN 315 [17]. The moisture was 6–14%, according to standard EN 322:1993 [18]. In the analyzed object, adhesive with standard EN 314-3 [19] was used, suitable for dry interior use only. The class of formaldehyde is E1, according to standard EN 717-2 [20]. Figure 1 presents the plywood structure together with three axes corresponding to the wood structure: L—longitudinal axis, parallel to the fiber (grain); R—radial axis, normal to the grain (perpendicular to the grain in the radial direction); T—tangential axis, perpendicular to the fibers, but tangent to the rings. The analyzed objects’ dimensions are presented in Table 1.

### 2.2. Finite Element Model of a Plywood Panel

The finite element model of the analyzed panel was built using a Midas NFX 2023 R1 preprocessor (Midas Information Technology Co. Ltd., Seongnam, Korea). A structured mesh for each layer constructed from eight-node, cubic, isoparametric finite CHEXA elements and six-node, five-walled, isoparametric CPENTA elements was used. The utilized finite elements were characterized by linear shape functions and three translational degrees of freedom in each node.

To describe the material properties of finite elements, the orthotropic material model MAT12 with a local coordinate system was used to model a specific orientation of individual layers. The strain–stress relationship of such a model for a three-dimensional stress state thus takes the following form [21]:

(1) $$\left[\begin{array}{c} \begin{array}{c} \begin{array}{c} \begin{array}{c} \varepsilon_{R}\\ \varepsilon_{L}\\ \varepsilon_{T} \end{array}\\ \gamma_{LR}\\ \gamma_{RT} \end{array}\\ \gamma_{TL} \end{array} \end{array}\right]=\left[\begin{array}{cccccc} \frac{1}{E_{L}} & \frac{-\upsilon_{RL}}{E_{R}} & \frac{-\upsilon_{TL}}{E_{T}} & 0 & 0 & 0\\ \ldots & \frac{1}{E_{R}} & \frac{-\upsilon_{TR}}{E_{T}} & 0 & 0 & 0\\ \cdots & \ldots & \frac{1}{E_{T}} & 0 & 0 & 0\\ \ldots & \ldots & \ldots & \frac{1}{G_{LR}} & 0 & 0\\ \ldots & \ldots & \ldots & \ldots & \frac{1}{G_{RT}} & 0\\ \ldots & \ldots & \ldots & \ldots & \ldots & \frac{1}{G_{TL}} \end{array}\right]\left[\begin{array}{c} \begin{array}{c} \begin{array}{c} \begin{array}{c} \sigma_{L}\\ \sigma_{R}\\ \sigma_{T} \end{array}\\ \tau_{LR}\\ \tau_{RT} \end{array}\\ \tau_{TL} \end{array} \end{array}\right],$$

where: $E_{i}$—modulus of elasticity in i direction, $G_{ij}$—shear modulus for the plane ij, $\varepsilon_{i}$—normal strain, deformation state component describing volumetric deformation in the direction i, $\gamma_{ij}$—shear strain, deformation state component describing form deformation in the plane ij, $\sigma_{i}$—normal stresses in the direction i, $\tau_{ij}$—tangential stresses in the in the plane ij, i,j—directions L, R, T. The first letter of the subscript refers to the direction of applied stress and the second letter to direction of lateral deformation. For example, $\upsilon_{LT}$ is the Poisson’s ratio for deformation along the tangential axis caused by stress along the longitudinal axis.

The three shear moduli denoted by $G_{LR}$, $G_{LT}$, and $G_{RT}$ are the elastic constants in the LR, LT, and RT planes, respectively. For example, $G_{LR}$ is the shear modulus based on shear strain in the LR plane and shear stresses in the LT and RT planes.

The shear modulus for two perpendicular material directions can be approximated according to the Saint–Venant’s equation [22]:

(2) $$G_{ij}={\left(\frac{1}{E_{i}}+\frac{\left(1+2\upsilon_{ji}\right)}{E_{j}}\right)}^{-1},$$

where: $\upsilon_{ji}$—Poisson’s ratio for deformation in the direction of axis j under load acting in the direction of axis i.

The connection between individual layers was carried out by coincidence of nodes. The model does not consider the material properties of the glue between the layers. Furthermore, the models analyzed in this study are unconstrained in order to minimize the impact of the unmodeled environment on their dynamic properties (also at a later stage of experimental testing).

The material properties describing the poplar are presented in Table 2. The wood properties were taken from [23,24] and from data sheets from plywood producer.

Next, a structural damping model was used to describe the damping properties of the subsequent layers, according to which the damping matrix C can be expressed as:

(3) C=iηK

where: K—stiffness matrix, i—imaginary unit, η—loss factor.

**Figure 2:**
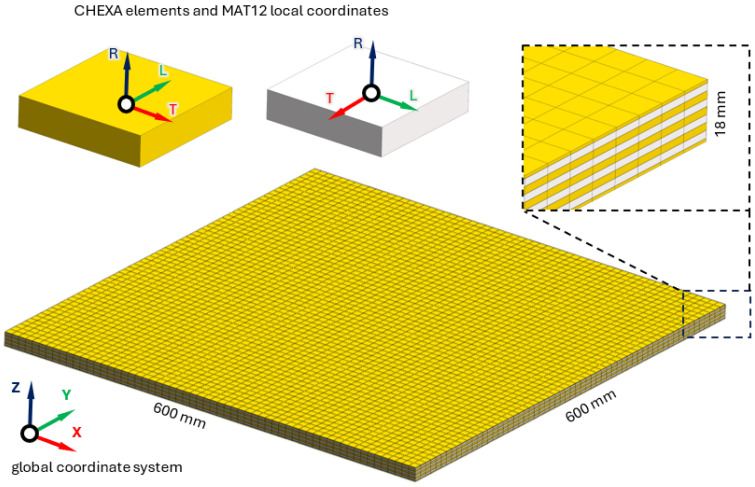
Finite element model of an 18 mm thick plywood panel.

The 18 mm tick panel model consisted of 32,400 elements and had 111,630 degrees of freedom, while the 27 mm tick panel model was built of 72,000 elements with a total number of 234,423 degrees of freedom. The established model for a plywood panel of 18 mm thickness is shown in Figure 2.

### 2.3. Experimental Testing

To verify the developed models, the experimental modal analysis in the form of an impact test was performed. To approximate the free boundary conditions, the analyzed panels were subsequently suspended on nylon strings, and the place of their attachment was selected in such a way as to best match the nodes of the analyzed mode shapes.

**Figure 3:**
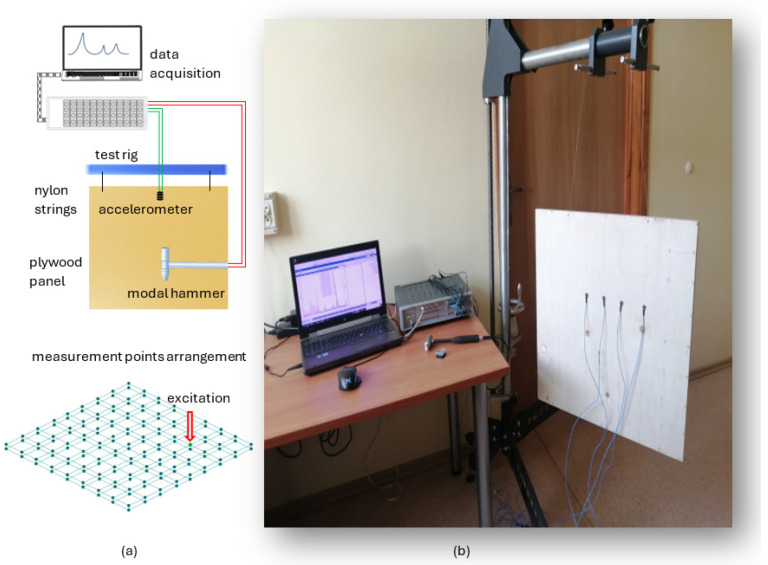
Modal analysis test stand with measurement points arrangement: schematic representation (**a**) and actual stand (**b**).

The panel was excited using the PCB 086C01 modal hammer (PCB Piezotronics, Depew, NY, USA) with a polymer tip in a direction perpendicular to the face sheet. The responses of the analyzed panel were measured in the same direction in 162 points (81 points each for the top face and bottom face) using the PCB 356A01 three-axis piezoelectric accelerometers (PCB Piezotronics, Depew, NY, USA). The measurements were performed simultaneously using 9 sensors, which resulted in 18 partial experiments—the sensors were moved, covering subsequent rows of the measurement grid. Data acquisition was performed using Scadas Mobile Vibco and Testlab 2019.1 software (Siemens AG, Munich, Germany). The estimation of frequency response functions was performed with the use of an H_1_ estimator. The remaining parameters of signal acquisition were as follows: sampling rate 4096 Hz, frequency resolution 0.5 Hz, number of averages 10. The test stand with measurement point arrangement is depicted in Figure 3.

As a result of the impact test conducted, 162 frequency response functions were determined, on the basis of which, using the Polymax algorithm [25,26], the parameters of the modal model of analyzed panels were estimated. The obtained modal models were validated using the MAC criterion, eliminating interdependent vectors in the mode shape (the limit value of 10% was assumed) [27].

## 3. Results

### 3.1. Experimental Verification

**Figure 4:**
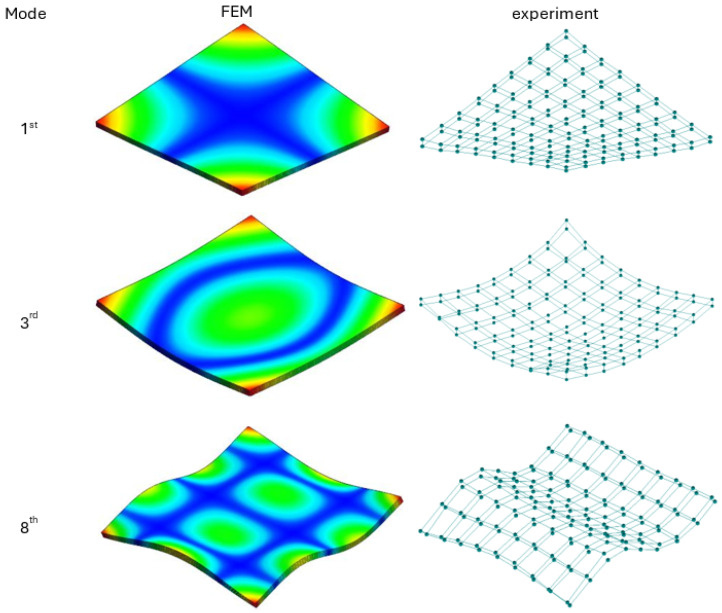
Selected mode shapes comparison—18 mm poplar plywood.

**Figure 5:**
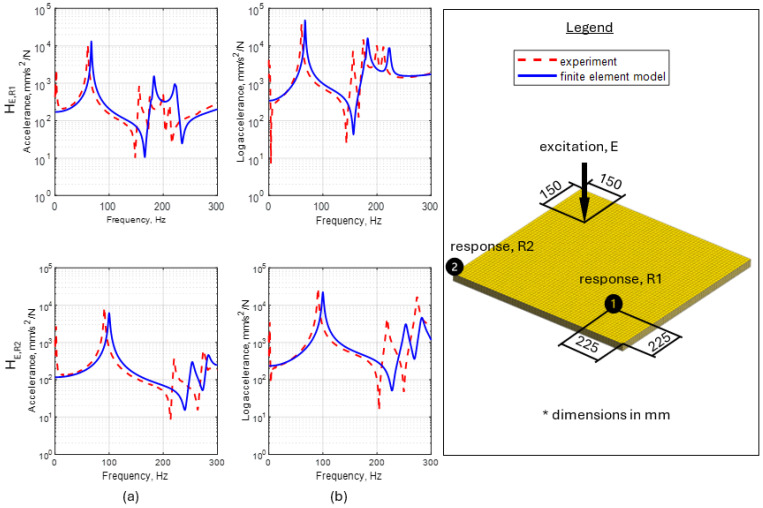
Comparison of calculated and experimentally determined accelerance functions for plywood 18 mm (**a**) and 27 mm (**b**).

The comparison of the values of natural frequencies for analyzed panels obtained numerically (using SOL103 solver) and experimentally supplemented with the relative error value is shown in Table 3. A comparison of selected modes is shown in Figure 4. Comparison of the accelerance functions determined computationally (using SOL108 solver) and experimentally is shown in Figure 5.

When analyzing the results obtained, it can be noted in the case of an 18 mm thick panel, all eight mode shapes were correctly identified in the analyzed frequency range. In the case of a 27 mm thick panel, the experiment failed to identify the eighth mode (with a total of nine determined from the model). When analyzing the accuracy of mapping the natural frequency values, it can be noted that in the case of a panel with a thickness of 18 mm, the maximum relative error for natural frequencies was 15.6% (for the sixth mode shape), an average of 9.6%. In the case of a 27 mm thick panel, the maximum error was also 15.6%, with an average of 11.0%. After analyzing the frequency response functions, it can be concluded that the obtained amplitude levels are satisfactorily accurate.

However, considering the overall accuracy of the model, it seems that the material values adopted from static tests are insufficient to obtain a reliable model of the dynamic properties of analyzed panels. Therefore, it was decided to fine-tune the model, which was preceded by an analysis of the sensitivity of its parameters.

### 3.2. Sensitivity Analysis

**Figure 6:**
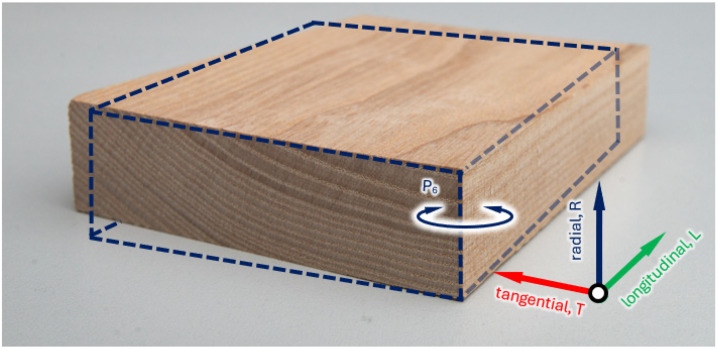
Schematic representation of P_6_ parameter definition.

A sensitivity analysis was performed to select appropriate decision variables for the next stage of model updating and to obtain insight into how changing individual parameters affects the dynamic properties of the model. It was realized for both plywood panels, i.e., 18- and 27 mm tick. Its practical implementation consisted of changing the values of selected model parameters in the range from 90% to 110% of their nominal value while observing how these changes affect the natural frequencies. The parameters adopted for the sensitivity analysis are shown in Table 4, while the results are depicted in Figure 6.

Parameters P_5_, P_6_ and P_7_ should be treated as variables indirectly describing the veneer production process, as they describe the angular deviation from the coordinate system of the wooden log, which is the raw material in the production of the veneer. To better visualize this, Figure 6 shows the idea behind those parameters on the example of P_6_.

**Figure 7:**
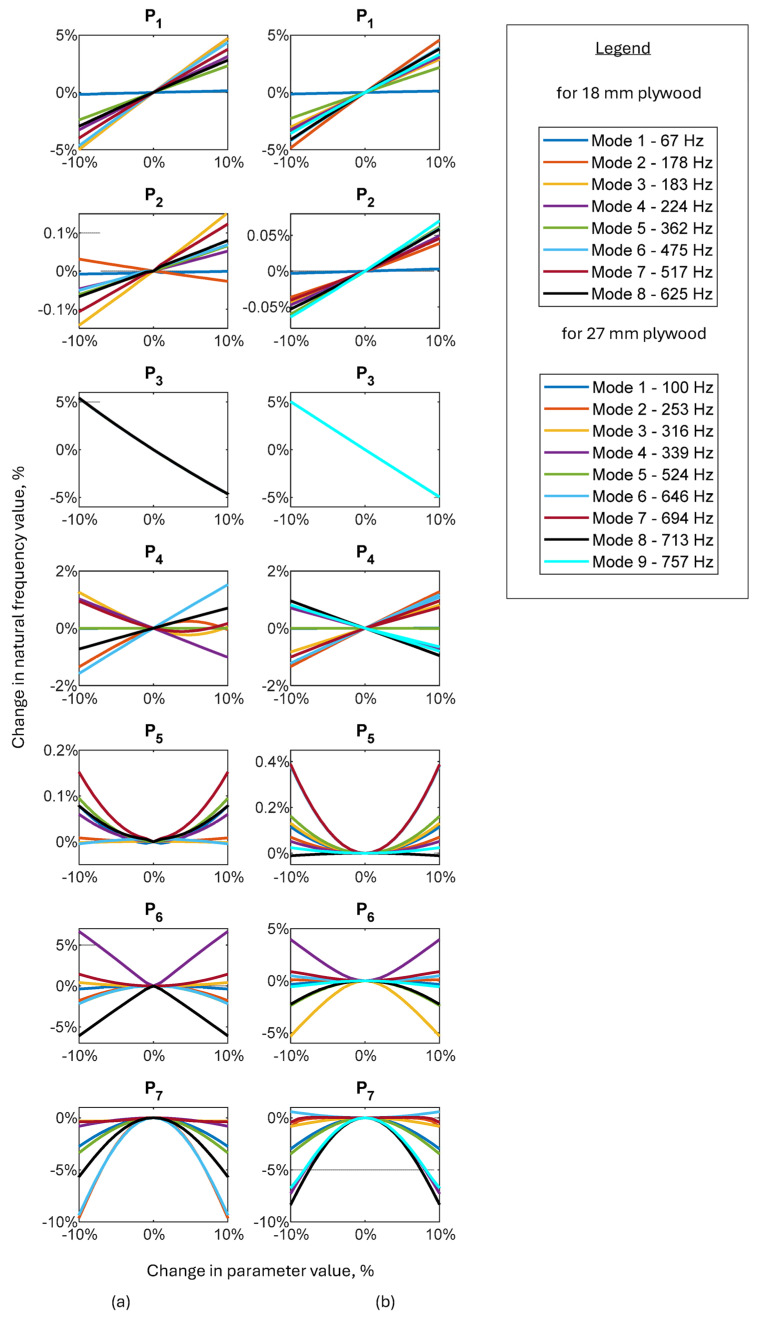
Sensitivity analysis results for 18 mm (**a**) and for 27 mm (**b**) plywood panels.

The results of the sensitivity analysis are presented in Figure 7.

After analyzing the obtained results, it can be seen that the greatest impact on the change in the natural frequency is a rotation of the material coordinate system about the tangential axis—parameter $P_{7}$. The nature of these changes is non-linear and applies to all modes in the analyzed frequency range. The greatest impact was observed for modes six and two for a panel with a thickness of 18 mm and for modes eight and four for a panel with a thickness of 27 mm, while the lowest impact was observed in both cases for modes seven and two.

The remaining parameters that have a significant impact on the values of natural frequencies are those related to the stiffness of the structure $P_{1}$ (simultaneous change of modulus of elasticity on three orthogonal directions), $P_{4}$ (thickness of the outer layers), and its mass—$P_{3}$ (material density). Here, the nature of the changes is mainly linear, which can be explained by a direct analogy to the stiffness-to-mass ratio, which reflects the natural frequency for a system with one degree of freedom.

The smallest impact on the natural frequency values was observed for the parameters $P_{2}$ (simultaneous change of Poisson ratio on three orthogonal directions) and $P_{5}$ (rotation of the material coordinate system about the longitudinal axis).

**Figure 8:**
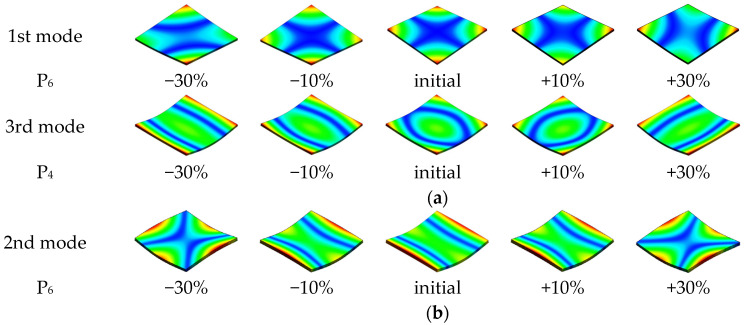
Mode shapes distortion for 18 mm thick (**a**) and 27 mm thick (**b**) plywood panels.

During the sensitivity analysis, not only results in changes in the natural frequency values were observed, but also changes in the mode shapes. Figure 8 shows changes in the mode shapes resulting from changes in parameter values (to better visualize the problem, the range of changes has been extended to ±30%).

Analyzing the results depicted in Figure 7, it can be seen that changing the angle between the layers relative to the radial axis first causes the mode for the 18 mm tick panel to change and, secondly, causes the mode for the 27 mm panel to change. 

Additionally, when changing the thickness of the outer layers while maintaining the total thickness of the plywood panel, the third mode for the 18 mm thick panel changes.

The changes observed for the analyzed panels, despite different causes, are ultimately characterized by the same effect: a change in the direction of the mode shape. Although this change may seem insignificant due to the symmetry of the panels, it may affect the model updating process (especially when high differences in parameter values after updating appear), hence the need for additional verification of the vibration mode for the already tuned model.

### 3.3. Model Updating

Considering the results obtained during sensitivity analysis, the following parameters were selected as decision variables in the model updating process: $P_{1}$ (simultaneous change of modulus of elasticity on three orthogonal directions), $P_{3}$ (density), and $P_{4}$ (thickness of outer layers). Taking a deeper look at the $P_{4}$ parameter, it should be noted that a simultaneous change in the thickness of the outer layers caused an analogous change in all the inner layers, although changing the layers’ thickness does not change the total thickness of the plywood.

Commenting on not considering the remaining parameters, the $P_{2}$ parameter (simultaneous change of Poisson ratio on three orthogonal directions) had a marginal impact on the change in the natural frequencies. Therefore, it was decided not to include it in the model updating process. Parameters $P_{5}$, $P_{6}$, and $P_{7}$ (rotation of the material coordinate system individual veneers about L, R, and T axes, respectively), although they provide important observations (discussed in Section 3), from a practical modeling point of view, they would be a clever solution to fine-tune the model rather than a carrier of reliable information about the structure. This is due to the fact that, typically, the manufacturing process for individual veneers is not as rigorous in terms of controlling angular position and, therefore, provides little traceable information in terms of timber cutting orientation. Moreover, since, as indicated earlier, the accelerance amplitude levels presented in Figure 5 show satisfactory agreement, it was also decided not to update the loss factor.

The process of identifying the model parameters is reduced to the task of minimizing the objective function, formulated as follows [28]:

(4) $$Q=\left[\begin{array}{c} {\hat{y}}_{p18}\\ {\hat{y}}_{p27} \end{array}\right]=\left[\begin{array}{c} {\left[{\ddot{y}}_{p18}^{exp}-{\ddot{y}}_{p18}^{fem}\right]}^{T}\left[{\ddot{y}}_{p18}^{exp}-{\ddot{y}}_{pp}^{fem}\right]\\ {\left[{\ddot{y}}_{p27}^{exp}-{\ddot{y}}_{p27}^{fem}\right]}^{T}\left[{\ddot{y}}_{p27}^{exp}-{\ddot{y}}_{p27}^{fem}\right] \end{array}\right]$$

where: ${\ddot{y}}_{p18}^{exp}$—experimentally determined accelerance function for 18 mm thick plywood panel, ${\ddot{y}}_{p27}^{exp}$—experimentally determined accelerance function for 27 mm thick plywood panel; ${\ddot{y}}_{p18}^{fem}$—accelerance function determined based on finite element model for 18 mm thick plywood panel, ${\ddot{y}}_{p27}^{fem}$—accelerance function determined based on finite element model for 27 mm thick plywood panel.

More precisely, during the model updating procedure, the difference between the frequency response functions determined numerically and experimentally is minimized simultaneously for 18 mm thick and 27 mm thick panels. This provides the possibility of universal (global) estimation of the structure parameters. A detailed description of the algorithm can be found in [28,29]. To solve the problem, MATLAB implemented interior point optimization algorithm was used [30]. The model-identified parameters are shown in Table 5, while a comparison of the natural frequencies is shown in Table 6.

**Figure 9:**
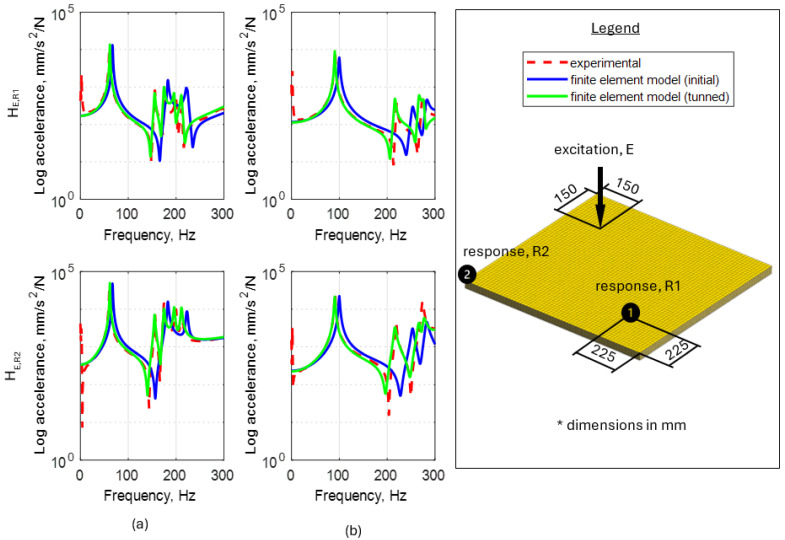
Comparison of frequency response functions before and after the model updating for 18 mm (**a**) and 27 mm (**b**) thick plywood panel.

Figure 9 shows a comparison of frequency response functions after model updating.

After the updating, the model was characterized by the same degree of compatibility of mode shapes (in the case of the 27 mm thick panel, the eighth mode was still not identified). However, significant changes took place regarding the values of the natural frequencies. After model updating, a decrease in the maximum relative error from 15.4% to 2.0% was observed, on average, from 9.5% to 0.7% in the case of the 18 mm thick panel. For the 27 mm thick panel, there was a decrease in maximum error from 15.5% to 2.6%, on average from 10.9% to 1.5%. Moreover, an improvement in the accuracy of mapping the frequency response functions was also observed.

Analyzing the obtained values of decision variables after updating, it can be noticed that most of them have ±20% variability (except for the thickness of the top and bottom layers, the variability of which is in the range of ±30%). 

### 3.4. Validation of Proposed Modeling Method

In the previous subsections, only unconstrained models of plywood panels were considered, obtaining—thanks to the proposed modeling method—a high agreement of calculations with experimental results. In reality, however, certain structures are built using those panels. Therefore, in order to prove the utility of the method and at the same time to validate it, a model of a cabinet was built, which was then subjected to experimental verification.

The cabinet main body has the dimensions of 400 × 600 × 800 mm. It consists of the following elements made of 18 mm thick laminated top plywood: two sides, a bottom shelf, and two rails. These elements are connected to each other using glued wooden pins. The cabinet door with a push lock handle is also made of 18 mm thick plywood and has a dimension of 776 × 396 mm. It is attached to the main body with two hinges. The main body is topped with a 27 mm thick double-sided laminated plywood panel measuring 420 × 630 mm. The cabinet does not have a back plate, because it is dedicated to vessels, in which furniture is attached to the wall using dedicated connectors.

**Figure 10:**
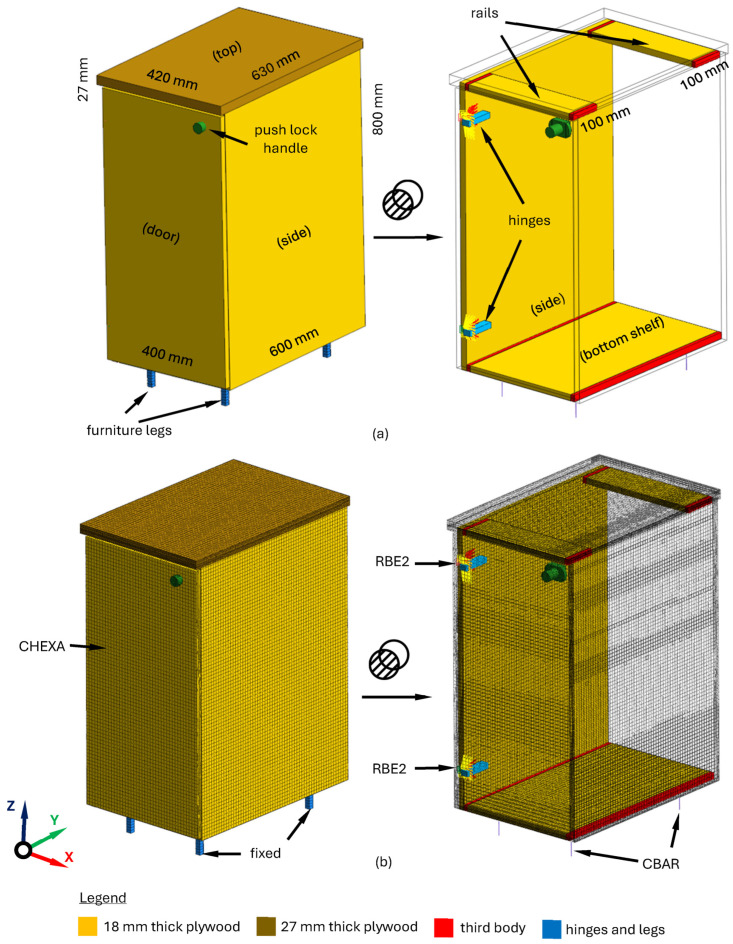
Geometrical structure (**a**) and finite element model (**b**) of cabinet.

The finite element model of the cabinet was built using the finite element model of a plywood panel established in Section 2.2. with material parameters identified in Section 3.3. and listed in Table 5. The connections of individual elements of the plywood cabinet were based on the third body contact model [31]. In the case of connecting the door with the main body, in order to reliably reproduce the connection nature (especially the area where it takes place), additional rigid finite elements (RBE2) were used [32]. The cabinet legs were modeled using a method presented in [33]. In total, the cabinet model consisted of 195,961 elements and had 675,201 degrees of freedom. Next, the model was constrained at the legs, where the actual cabinet touches the floor. The cabinet structure, together with the finite element model established, is depicted in Figure 10. 

**Figure 11:**
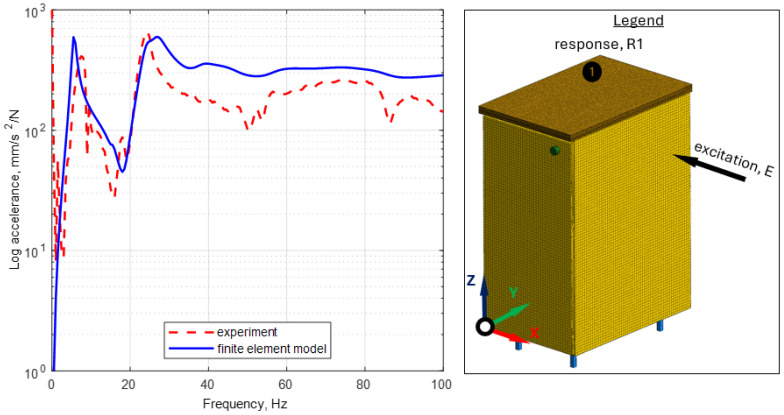
Comparison of frequency response functions for cabinet model MES and experimentally determined.

To verify the developed model, an experimental modal analysis was carried out in the form of an impact test using the stand presented in Section 2.3. Table 7 presents the experimental verification of natural frequencies, while Figure 11 presents experimental verification for exemplary frequency response function. 

After analyzing the results obtained, it can be noted that eleven mode shapes were correctly identified in the analyzed frequency range. In the case of the accuracy of mapping the natural frequency values, it can be noted that the maximum relative error was 17.9% (for the second mode shape—a rocking mode, in which mapping accuracy depends mostly on leg characteristics), an average of 6.6%. Analyzing the frequency response functions, it can be concluded that the obtained amplitude levels are satisfactorily accurate.

## 4. Discussion

The methodology for modeling the dynamic properties of plywood presented in the article is able to effectively predict its dynamic properties, which has been confirmed by experimental verification.

The analysis presented in the paper indicates that building a finite element model based on the values of material parameters derived from static tests gives significant discrepancies in the values of the natural frequencies. Hence, to achieve high accuracy, it seems necessary to use a model updating approach, which allowed for a decrease in the maximum relative error from 15.5% to 2.0%, on average from 9.5% to 0.7% in the case of the 18 mm thick panel and, respectively, from 15.5% to 2.6%, and from 10.9% to 1.5% for 27 mm thick panel. Additionally, an improvement in the accuracy of mapping the accelerance was achieved. 

After analyzing the identified values of model parameters, it can be noticed that most of them have ±20% variability (except for the thickness of the top and bottom layers, the variability of which is in the range of ±30%). Due to significant differences, these values may raise some concerns, although taking into account the characteristics of the material itself [9], the veneer production process [34], as well as other studies reporting on wood parameters uncertainty [35], these differences seem to be acceptable.

The proposed modeling method was validated using the developed models with identified material parameters to predict the dynamic properties of the cabinet. Although the proposed validation is not direct in nature, as it considers additional elements in the model, such as hinges and connections between panels and legs, it indicates the usefulness of the modeling methodology. When analyzing the structural mode shapes (i.e., mainly dependent on the dynamic properties of plywood panels), high accuracy of mapping can be noticed (the average relative difference for natural frequencies is 4.1%).

Although the parameters $P_{5}$, $P_{6}$, and $P_{7}$ related rotation of the material coordinate system individual veneers about L, R, and T axes, respectively, were not considered in the model updating process, their sensitivity analysis provides some information that may prove useful in a broader perspective regarding the modeling of wooden structures. Changes in the values of these parameters have a significant impact on the dynamic properties of the modeled panels; what is even more problematic is that the nature of these changes is non-linear and applies to all modes identified in the analyzed frequency range. This implies that, regardless of the diligence of the research and the effort put into modeling, the value of material parameters characterized by high variability and low traceability resulting from the nature of the wood and the production process, respectively, is a key obstacle to obtaining repeatable, accurate modeling results.

## 5. Conclusions

Modeling the dynamic properties of wood products and wood-based composites is a difficult task due to the naturally growing structure of wood, its moisture-dependent material properties, and production process conditions. Despite these difficulties, the properties of wood make it a construction material eagerly used by designers in many industries. This forces the improvement of the existing and the development of new computational methods to predict the operational properties of structures made of wooden materials.

In an attempt to contribute to this subject, the article presents the finite element modeling of the dynamic properties of plywood panels. The dynamic properties analyzed were natural frequencies, mode shapes, and accelerance. Based on the developed model consisting of individual layers of wood, which were discretized using three-dimensional finite elements with orthotropic material formulation, the modeling method proved effective in numerically predicting listed dynamic characteristics.

Developed plywood models were then used to model a cabinet to prove their utility. The experimental verification of the model showed a satisfactory agreement with its real counterpart, confirming the validity of the assumptions and modeling procedure.

The main limitation of the study is the lack of tests for plywood made from different production batches and different wood species. Such research would make it possible to obtain quantitative results that could support the hypothesis about the ambiguity of the solution related to the nature of the wood and the production process. They would also constitute a validation of the modeling methodology in a much broader perspective than that presented in the article. Future work should, therefore, focus on trying to solve the problems raised. Moreover, it seems reasonable to introduce the decoupling of the modulus of elasticity in the model updating procedure so that each component, i.e., $E_{L}$, $E_{R}$, $E_{T}$, constitutes a separate decision variable.

## Figures and Tables

**Table 1:** Dimensions of analyzed panels.

Data	Object	
	Plywood 18 mm	Plywood 27 mm
Dimensions, mm	600 × 600	600 × 600
Thickness, mm	18 ± 0.3	27 ± 0.3
Number of layers	9	11
Thickness of the plies		
Top layer, mm	0.95 ± 0.05	1.00 ± 0.05
Internal layers, mm	2.30 ± 0.05	3.00 ± 0.05
Bottom layer, mm	0.95 ± 0.05	1.00 ± 0.05

**Table 2:** Material data for poplar.

Parameter	Value	Unit
Modulus of elasticity along longitudinal axis, $E_{L}$	10,900	MPa
Modulus of elasticity along radial axis, $E_{R}$	1003	MPa
Modulus of elasticity along tangential axis, $E_{T}$	469	MPa
Poisson ratio, $\upsilon_{LT}$	0.392	–
Poisson ratio, $\upsilon_{TR}$	0.329	–
Poisson ratio, $\upsilon_{RL}$	0.030	–
Shear modulus, $G_{LT}$	752	MPa
Shear modulus, $G_{TR}$	120	MPa
Shear modulus, $G_{RL}$	818	MPa
Density, ρ	450	kg/m^3^
Damping ratio	0.023	–

**Table 3:** The results of experimental verification of the natural frequencies of the models.

Mode Shape	Natural Frequency, Hz		Relative Error, %
	Experiment	Finite Element Model	
Plywood 18 mm			
1.	60.3	67.2	11.3
2.	155.0	177.8	14.7
3.	173.9	182.6	5.0
4.	210.9	223.6	6.0
5.	335.3	362.4	8.1
6.	411.1	474.6	15.4
7.	493.6	516.8	4.7
8.	563.8	625.5	10.9
		On average	9.5
Plywood 27 mm			
1.	91.0	100.0	9.9
2.	219.1	253.0	15.5
3.	274.0	316.3	15.4
4.	324.0	338.8	4.6
5.	485.1	523.9	8.0
6.	559.3	645.8	15.5
7.	608.4	693.7	14.0
8.	not identified	713.2	–
9.	724.4	756.8	4.5
		On average	10.9

**Table 4:** Parameters adopted for the sensitivity analysis.

Designation	Description
P_1_	modulus of elasticity, simultaneous change of $E_{L}$, $E_{R}$, $E_{T}$
P_2_	Poisson ratio, simultaneous change of $\upsilon_{LT}$, $\upsilon_{TR}$, $\upsilon_{RL}$
P_3_	material density
P_4_	thickness of the top and bottom layers (veneers), maintaining a constant total thickness of the panel
P_5_	rotation of the veneer material coordinate system around the longitudinal axis
P_6_	rotation of the veneer material coordinate system around the radial axis
P_7_	rotation of the veneer material coordinate system around the tangential axis

**Table 5:** Comparison of the decision variables values before and after the model updating.

Parameter		Unit	Initial Value	Identified Value	Relative Difference, %
$P_{1}$	Modulus of elasticity along longitudinal axis, $E_{L}$	MPa	10,900	9240	15.2
	Modulus of elasticity along radial axis, $E_{R}$	MPa	1003	850.08	
	Modulus of elasticity along tangential axis, $E_{T}$	MPa	469	397.32	
$P_{3}$	Density, ρ	kg/m^3^	450	455	1.1
$P_{4}$	Thickness of the top and bottom layers (veneers)	mm	0.95	0.67	29.5
	Thickness of the internal layers (veneers)	mm	2.30	2.38	3.5

**Table 6:** The results of experimental verification of the natural frequencies of the models.

Mode Shape	Experiment, Hz	Finite Element Model (Initial Parameters), Hz	Relative Error, %	Finite Element Model (Identified Parameters), Hz	Relative Error, %
Plywood 18 mm					
1.	60.3	67.2	11.4	61.5	2.0
2.	155.0	177.8	14.7	155.0	0.0
3.	173.9	182.6	5.0	174.5	0.3
4.	210.9	223.6	6.0	211.1	0.1
5.	335.3	362.4	8.1	331.7	1.1
6.	411.1	474.6	15.4	412.8	0.4
7.	493.6	516.8	4.7	489.4	0.9
8.	563.8	625.5	10.9	559.5	0.8
		On average	9.5	On average	0.7
Plywood 27 mm					
1.	91.0	100.0	9.9	90.5	0.5
2.	219.1	253.0	15.5	216.2	1.3
3.	274.0	316.3	15.4	276.4	0.9
4.	324.0	338.8	4.6	315.5	2.6
5.	485.1	523.9	8.0	474.2	2.2
6.	559.3	645.8	15.5	554.2	0.9
7.	608.4	693.7	14.0	601.0	1.2
8.	not identified	713.2	–	670.4	-
9.	724.4	756.8	4.5	707.7	2.3
		On average	10.9	On average	1.5

**Table 7:** Experimental verification of mode shapes for cabinet.

Mode Shape	Mode Type	Experiment, Hz	Finite Element Model, Hz	Relative Error, %
1.	rocking	6.8	5.5	17.9
2.	rocking	18.2	15.3	15.9
3.	rocking	22.3	24.1	8.1
4.	structural	27.3	26.4	3.3
5.	structural	46.1	47.3	2.6
6.	structural	49.3	42.1	14.4
7.	structural	57.4	56.3	1.7
8.	structural	70.9	69.1	2.5
9.	structural	84.6	83.9	0.8
10.	structural	91.3	95.2	4.3
11.	structural	97.3	98.8	1.5
			On average	6.6

## Data Availability

The original contributions presented in the study are included in the article, further inquiries can be directed to the corresponding author.
